# Supplementary material for: Visualization of Bacterial Protein Complexes Labeled with Fluorescent Proteins and Nanobody Binders for STED Microscopy
Source: Int J Mol Sci. 2019 Jul 10;20(14):3376. doi: 10.3390/ijms20143376 (PMC6678925; doi:10.3390/ijms20143376)
Supplement: Supplementary file 1 [file ijms-20-03376-s001.pdf]

Supplementary Materials

Visualization of bacterial protein complexes labeled with fluorescent proteins and nanobody binders for STED microscopy

Kimberly Cramer[1,2,*], Anna-Lena Bolender[3,*] , Iris Stockmar[1,2], Ralf Jungmann[1,2], Robert Kasper[3,#], Jae Yen Shin[1,2,#]

[1]Max Plank Institute of Biochemistry, 82152 Martinsried, Munich, Germany.

[2]Faculty of Physics and Center for Nanoscience, Ludwig Maximilian University, 80539 Munich, Germany.

[3]Max Plank Institute of Neurobiology, 82152 Martinsried, Munich, Germany.

*Equally contributed authors: Kimberly Cramer and Anna-Lena Bolender.

[#]Correspondence: rkasper@neuro.mpg.de (R.K.) jshin@biochem.mpg.de (J.Y.S.)

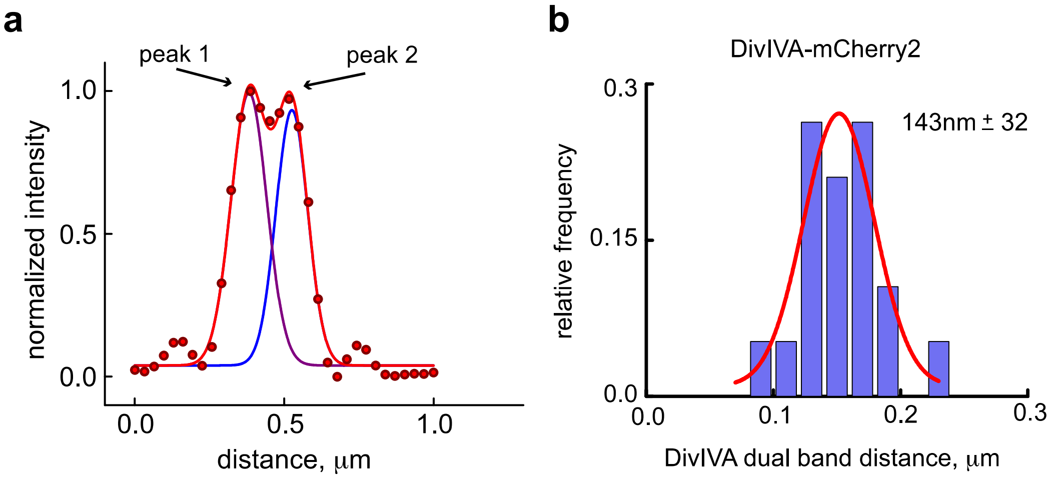

Figure S1. Histogram of distance between the DivIVA dual band in SIM images. Intensity profiles of the mCherry2 fluorescent signal along the longitudinal axis of cells from *B. subtilis* cells expressing DivIVA-mCherry2 were obtained using Fiji. **(a)** To extract the center position of each band along the longitudinal axis, the sum of two Gaussian functions were fitted (solid line) to this intensity profile (circles), using the software OriginPro 9.1G. Then, the values for the two center parameters (peak 1 and peak 2) of the Gaussians were subtracted to calculate their distance. **(b)** Mean and standard deviation of the distance distribution are indicated in the histogram.

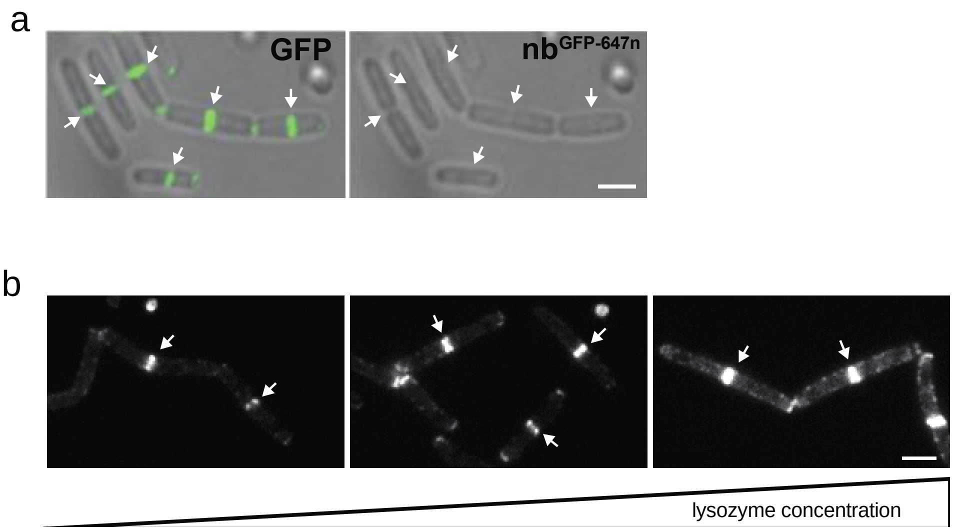

Figure S2. Effect of lysozyme treatment on nanobody binder entry into *B. subtilis*. (a) Overlay of bright field (gray) and fluorescence signal from GFP (left) in green, and NB^{GFP}-Atto647n (right) in *B. subtilis* cells expressing DivIVA-GFP (1803) treated with 0 mg/ml of lysozyme. Arrows indicate division septa where DivIVA-GFP is localized. Scale bar 2 μm. **(b)** Fluorescence signal from NB^{RFP}-Atto647n in *B. subtilis* cells expressing DivIVA-PAmCherry (BHF1) treated with 0.04, 0.2, and 0.4 mg/ml of lysozyme, left to right. Arrows indicate NB^{RFP}-Atto647n signal at division septa. Scale bar 3 μm. Images were taken with a diffraction-limited microscope. In (a), GFP imaging was performed with an exposure of 0.2s and ND filter = 32%, and NB^{GFP}-Atto647n imaging was performed with an exposure time of 0.5s and ND filter = 32%. In (b), exposure was 0.2s and ND filter = 50% for NB^{GFP}-Atto647n imaging.

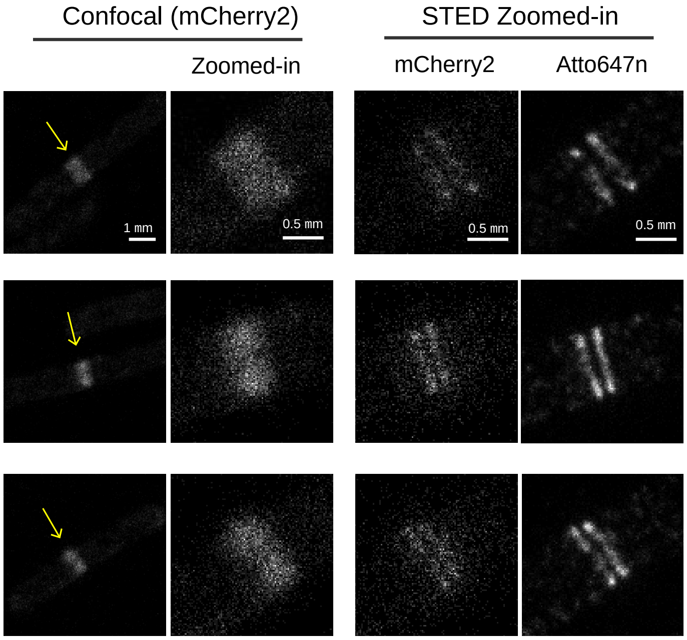

Figure S3. Comparison of mCherry2 and Atto647n for STED imaging. *B. subtilis* expressing DivIVA-mCherry2 treated with the immunolabeling protocol using the binder NB^{RFP}-Atto647n (as described in Material and Methods) were imaged with STED microscopy. Each row displays a single cell. Yellow arrows indicate DivIVA localization at division septa, and their corresponding zoomed-in confocal and STED images are shown in the Middle and Right columns.

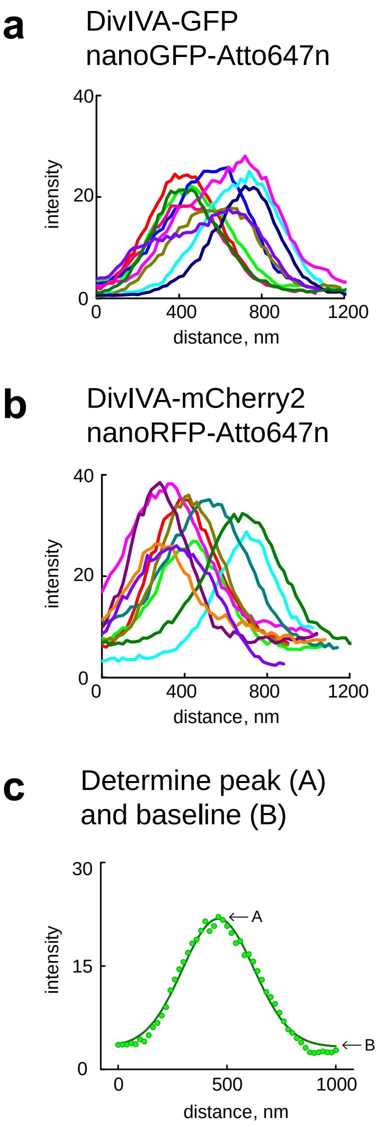

Figure S4. Determination of signal-to-background ratio. (a, b) Intensity profiles of the fluorescent signal from the fluorophore conjugated nanobody-along the longitudinal axis of cells from *B. subtilis* cells expressing either DivIVA-GFP or DivIVA-mCherry2. Each line represents the signal distribution from one cell, which was obtained by drawing a line along the longitudinal axis of cells using ImageJ. **(c)** Each intensity profile (circles) was fitted with a single peak gaussian function (continuous line), peak height at center point (A) and baseline (B) were obtained using the software OriginPro 9.1G.

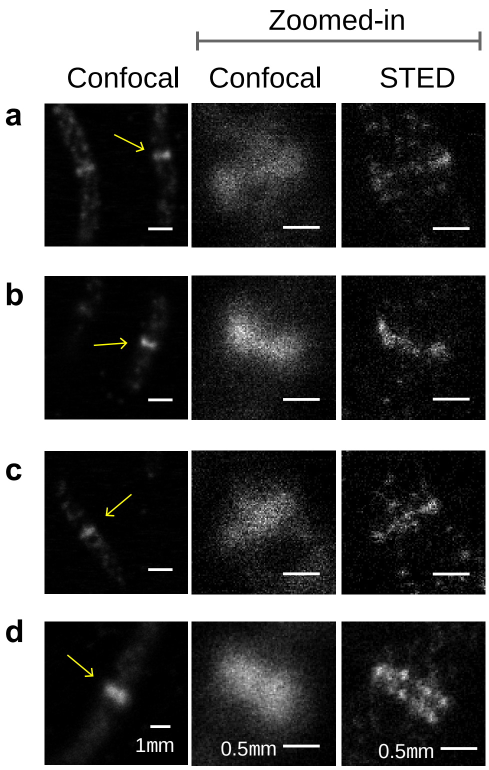

Figure S5. Visualization of FtsZ in *Escherichia coli* with STED microscopy. Strain EC484 containing GFP tagged FtsZ was visualized using NB^{GFP}-Atto647n via confocal and STED microscopy. Four examples are shown in **(a-d).** Confocal images in the *Left column* show FtsZ localization in single cells. Yellow arrows indicate FtsZ localization at division septa for **(a-d)**, and their corresponding zoomed-in confocal and STED images are shown in the *Middle* and *Right columns*, respectively.

Table S1. Cellular background for *B. subtilis* expressing mCherry2 and GFP fusion protein.

FP	Fused to	Signal to background ratio (A/B)	Cellular Background (B/A*100)
GFP	DivIVA	2.6	40 %
mCherry2	DivIVA	2.9	35 %

Signal-to-background ratio and cellular background were calculated as described in the main text and SM Figure 3. n = 31.

Table S2: Properties of dyes and fluorescent proteins used for STED microscopy in this study. Lists fluorescent proteins and dye molecules with net charge, hydrophobicity, molecular weight, $\lambda_{excitation}$ and $\lambda_{emission}$.

Dye	Net charge (after coupling)	Hydrophobicity	Molecular weight (g/mol)	λ_{abs}, nm	λ_{fl}, nm	*REF
Atto595	-1	Very hydrophilic	1,389	603	626	Atto-tec
Atto647n	+1	Moderately hydrophilic	843	646	664	Atto-tec
Star600	0 (zwitterionic)	hydrophilic	880.9	604	623	Abberior
Star635p	-3	hydrophilic	1,030.8	638	651	Abberior
mCherry2	n.a.	n.a.	26,700	585	610	[1]

*obtained from dye information pages provided by Atto-tec of Abberior companies

SM Materials and Methods

Table S3: Lists bacterial strains used in this study.

Name	Genotype	Species	Citation
1803	*divIVA::(PdivIVA*-gfp *divIVA* $^{+}$ cat*)*	*B. subtilis*	[2]
BHF3	*divIVA*::divIVA-mCherry2 spec	*B. subtilis*	This study
BHF1	*divIVA*::divIVA-PAmCherry spec	*B. subtilis*	[3]
EC484	*P* $_{208}$-*ftsZ-gfp leu*::Tn*10*	*E. coli*	[4]

Table S4: STED sample preparation reagents and conditions. Lists of nanobodies binders used in this study, the targets the binders imaged, the dilutions employed, Figures in the Main Text where imaging using each binder appears, and their Cat. No.

Binder	Target imaged	Dilution	Corresponding Figure	Cat. No. *
NB^{GFP}-atto594	DivIVA-GFP	1:250	3, 4	gba594
NB^{GFP}-atto647n	DivIVA-GFP	1:250	2, 3, 4	gba647n

NB^{GFP}-star635p	DivIVA-GFP	1:250	3, 4	gbas635p
NB^{RFP}-atto594	DivIVA-mCherry2	1:250	3, 4	rba594
NB^{RFP}-atto647n	DivIVA-mCherry2	1:250	2, 3, 4	rba647n
NB^{RFP}-star600	DivIVA-PAmCherry	1:250	4	n/a$

*Cat. No. for Chromotek GmbH products.

$ Gifted

References

1. Shen, Y.; Chen, Y.; Wu, J.; Shaner, N. C.; Campbell, R. E. Engineering of mCherry variants with long Stokes shift, red-shifted fluorescence, and low cytotoxicity. *PLoS One* **2017**, *12*, 1–14.
2. Hamoen, L. W.; Meile, J.-C.; de Jong, W.; Noirot, P.; Errington, J. SepF, a novel FtsZ-interacting protein required for a late step in cell division. *Mol Microbiol* **2006**, *59*, 989–999.
3. Stockmar, I.; Feddersen, H.; Cramer, K.; Gruber, S.; Jung, K.; Bramkamp, M.; Shin, J. Y. Optimization of sample preparation and green color imaging using the mNeonGreen fluorescent protein in bacterial cells for photoactivated localization microscopy. *Sci Rep* **2018**, 1–11.
4. Weiss, D. S.; Chen, J. C.; Ghigo, J. M.; Boyd, D.; Beckwith, J. Localization of FtsI (PBP3) to the septal ring requires its membrane anchor, the Z ring, FtsA, FtsQ, and FtsL. *J Bacteriol* **1999**, *181*, 508–520.
